# Supplementary material for: Smooth Interpolating Curves with Local Control and Monotone Alternating Curvature
Source: Comput Graph Forum. 2022 Oct 6;41(5):25–38. doi: 10.1111/cgf.14600 (PMC9827861; doi:10.1111/cgf.14600)
Supplement: Supplementary file 1 — Supplement Material [file CGF-41-25-s001.zip › Local-Smooth-Interpolating-MonoCurvature/extern/clothoids/docs/api-cpp/variable_a00119_1ab7ae60f08d4dcaa2b289bdd7bb3dc4ee.html]

Variable G2lib::m\_1\_sqrt\_pi — Clothoids v2.0.9

### Navigation

- index
- toc
- next
- previous
- Clothoids »
- C++ API »
- Variable G2lib::m\_1\_sqrt\_pi

# Variable G2lib::m\_1\_sqrt\_pi¶

- Defined in File G2lib.cc

## Variable Documentation¶

real\_type const G2lib::m\_1\_sqrt\_pi = 0.564189583547756286948079451561¶
:   \( 1/\sqrt{\pi} \)

### Quick search

### Table of Contents

- Matlab Interface Manual
- C++ API
- MATLAB API

«
hide menu

menu
sidebar
»

### Navigation

- index
- toc
- next
- previous
- Clothoids »
- C++ API »
- Variable G2lib::m\_1\_sqrt\_pi

© Copyright 2021, Enrico Bertolazzi and Marco Frego.
Created using Sphinx 4.2.0.
